# New Poverty Line and Growth Chart Bring Forth Sharp Inequalities in the Indian Population

**DOI:** 10.4103/0970-0218.55266

**Published:** 2009-07

**Authors:** Sanjiv Kumar Bhasin

**Affiliations:** Department of Community Medicine, University College of Medical Sciences and GTB Hospital, Delhi, India. E-mail: sk1bhasin@gmail.com

The last few months have seen a fierce debate over the World Bank's new poverty estimates for India,(1) coming close on heels of the new WHO growth charts, to grade the nutritional status of children.(2) On the basis of employing a new poverty line of $1.25 per day at 2005 Purchasing Power Parity (PPP), made available on account of new information on PPP exchange rates, due to the efforts of the International Comparison Program, the World Bank estimates that 41.6% of India's population (approximately 455 million) is now living below the poverty line. This is much above the 24% based on the earlier criteria of $1 per day. If these statistics are not bad enough, the Asian Development Bank (ADB) has estimated an even higher number of poor (ranging between 622 and 740 million in 2005) using a higher Asian poverty line of $1.35 per day per person.(3) These statistics are more than double the poverty estimates of 28% by the Planning Commission of India,(4) which utilized a poverty line criteria based on NSSO's 61^st^ round survey of Rs.356 per capita for rural families and Rs.540 per capita for urban families. This poverty line criterion is based on per capita expenditure level at which an average per capita calorie intake is 2,400 and 2,100 calories for rural and urban areas, respectively. Obviously, these extremely high numbers of poor in India have generated much disbelief, since the country is priding itself on extremely high GDP growth rates in the new millennium.

The Government of India has accepted the new WHO child growth standards,(5) based on the WHO Multicenter Growth Reference Study, which now estimates severe malnutrition (i.e., below -3 standard deviation of median) in children between 1 and 3 years of age to be 15.6%, much above the current estimate of 4.2%, based on the Indian Academy of Pediatrics (IAP) classification, and 13.7% in children between 3 and 5 years of age, again way higher than the current estimate of 2.9%. These lines and charts have drawn criticism from among a group of economists and policy makers, not the least on account of using different, albeit a new criteria base, even though they are updated and based on better scientifically available information. In fact these depressing statistics seem realistic and may actually be like correctives if one takes into account some other simultaneously available reports on nutritional status and expenditure of our population. Therefore, instead of lamenting and denying these statistics, it may be worthwhile to focus on taking urgent steps to bring down these gross income and health disparities among our populace.

The fact that poverty lines (based either on income or calorie criteria) have very strong if not a direct association with the nutritional status of children (and adults also) is all too well known. Thus while virtually every second young child in the country is undernourished, NFHS-3 data shows a whopping 79% of the children in the age group 6 to 35 months to be anemic. According to the National Commission for Enterprise in the Unorganized Sector(6) commissioned the Arjun Sengupta Report (2007) on the Conditions of Work and Promotion of Livelihood in the Unorganized Sector, an overwhelming 836 million people in India live on a per capita consumption of less than Rs 20 a day, based on government data for the period between 1993 and 1994 and 2004 and 2005. Late last year India ranked 66 out of 88 countries on the 2008 Global Hunger Index (GHI), released by the International Food Policy Research Institute.(7) This composite index was based on an average of three major indicators, that is, child mortality rates, child malnutrition rates, and the proportion of calorie-deficient population. That there has been an unabated decline (increasing to as much as 3.2% annually between 1991 and 2003) in per capita pulse (besides many other basic food items) consumption in the Indian diet is virtually a long forgotten story.(8)

Thus basically, the problem is one of not having enough income with a vast majority of population to meet the daily food requirement. Statistics aside, clearly high GDP growth rates have not translated into any trickle down effect for huge sections of our population. There is a crying need for rapid and sustained growth, but more importantly economic growth must be inclusive growth, one that addresses gross disparities, generates employment and at least ensures food and nutrition security. It calls for an urgency to address the issue of reviving the stagnant agriculture sector to increase food production and incomes of Below Poverty Line families. The contribution of the agriculture sector to GDP has declined from 55.4% in 1950 – 1951 to 22.8%, whereas, the proportion of the agriculturist to the agricultural workers remains more or less the same.(9) The growth of employment in the industrial and service sectors is also very low. Not even half of the seven million workforce entering the job market every year is getting employed.(10) There is need for a faster and robust development of the agricultural sector, to provide both immediate employment and basic food (and caloric) requirement. Also there is a need to put in place a more enlarged and efficiently working public distribution system, along with a responsive, regulated, accountable, and functioning public health system, to take care of the nutritional and health needs of vast masses of poor and marginalized people. The State must assume major responsibility and increase investment in agriculture, health and other social welfare sectors substantially, to make some dent in the gross income and health inequalities.
